# Genetic screen for factors mediating PIN polarization in gravistimulated *Arabidopsis thaliana* hypocotyls

**DOI:** 10.1111/tpj.14301

**Published:** 2019-04-10

**Authors:** Hana Rakusová, Huibin Han, Petr Valošek, Jiří Friml

**Affiliations:** ^1^ Institute of Science and Technology (IST) Austria 3400 Klosterneuburg Austria

**Keywords:** gravitropism, auxin transport, PIN proteins, cell polarity, *Arabidopsis thaliana*, forward genetic screen, *SCARECROW*, actin cytoskeleton.

## Abstract

Gravitropism is an adaptive response that orients plant growth parallel to the gravity vector. Asymmetric distribution of the phytohormone auxin is a necessary prerequisite to the tropic bending both in roots and shoots. During hypocotyl gravitropic response, the PIN3 auxin transporter polarizes within gravity‐sensing cells to redirect intercellular auxin fluxes. First gravity‐induced PIN3 polarization to the bottom cell membranes leads to the auxin accumulation at the lower side of the organ, initiating bending and, later, auxin feedback‐mediated repolarization restores symmetric auxin distribution to terminate bending. Here, we performed a forward genetic screen to identify regulators of both PIN3 polarization events during gravitropic response. We searched for mutants with defective PIN3 polarizations based on easy‐to‐score morphological outputs of decreased or increased gravity‐induced hypocotyl bending. We identified the number of *hypocotyl reduced bending* (*hrb*) and *hypocotyl hyperbending* (*hhb*) mutants, revealing that reduced bending correlated typically with defective gravity‐induced PIN3 relocation whereas all analyzed *hhb* mutants showed defects in the second, auxin‐mediated PIN3 relocation. Next‐generation sequencing‐aided mutation mapping identified several candidate genes, including *SCARECROW* and *ACTIN2*, revealing roles of endodermis specification and actin cytoskeleton in the respective gravity‐ and auxin‐induced PIN polarization events. The hypocotyl gravitropism screen thus promises to provide novel insights into mechanisms underlying cell polarity and plant adaptive development.

## Significance Statement

Plant gravitropism involves two distinct cellular polarizations of PIN3 auxin transporter to redirect intercellular auxin fluxes for the bending response. Here we identified a number of Arabidopsis mutants defective specifically in the PIN3 polarization events, establishing that gravity‐induced polarization is required for bending initiation and auxin feedback‐mediated second polarization for its termination. Molecular analysis of selected mutants revealed a crucial role of SCARECROW‐mediated endodermis specification and actin cytoskeleton in these polarization events during gravitropism.

## Introduction

Plants exhibit developmental plasticity to adjust to the changing environmental conditions. Phototropism and gravitropism are two examples of how plants flexibly adapt their growth in response to light and gravity, respectively (Harmer and Brooks, 2018). Directional transport‐mediated asymmetric distribution of the plant hormone auxin (Luschnig *et al*., 1998; Friml *et al*., 2002) is the key trait in response to light or gravity stimuli. Auxin accumulates at the lower side of roots and hypocotyls during gravity stimulation or at the shaded side of hypocotyls in case of unilateral light stimulation (Kleine‐Vehn *et al*., 2010; Ding *et al*., 2011; Rakusová *et al*., 2011), ultimately leading to asymmetric growth and organ bending (de Wit *et al*., 2016; Su *et al*., 2017).

**Figure 1:**
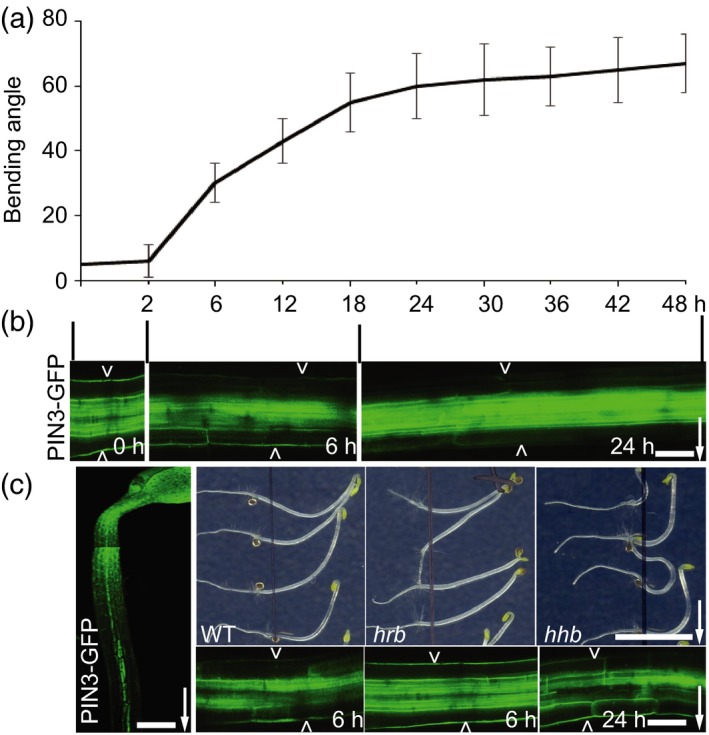
Mutant screen for PIN3 polarization defects in hypocotyl gravitropism. (a) Arabidopsis hypocotyl bending kinetics. Three‐day‐old etiolated seedlings were gravistimulated and bending angle was measured at indicated time intervals (h), data referred from Rakusová *et al*. (2016); *N *> 20 seedlings. (b) PIN3‐GFP polarization at different stages of gravitropic response. Before gravity stimulation, PIN3‐GFP is symmetric at both sides of endodermal cells. Starting at 2 h, PIN3‐GFP is enriched at the lower side of endodermal cells. After approximately 18 h, PIN3‐GFP at the lower hypocotyl side starts to disappear from the outer sides of endodermal cells restoring the symmetry between the upper and lower sides of the hypocotyl. Pictures were taken at 0, 6 and 24 h after gravity stimulation. Scale bar: 20 μm. Arrow indicates gravity direction. (c) Design of the forward genetic screen for novel PIN3 polarity regulators. Ethyl methylsulfonate (EMS)‐mutagenized 3‐day‐old etiolated *PIN3::PIN3‐GFP* seedlings were gravity stimulated for 6 or 24 h. Seedlings with reduced bending (*hrb* mutants) or hyperbending (*hhb* mutants) response were selected as candidates. PIN3‐GFP relocation after gravity stimulation was analyzed in selected mutants. Typically, in the *hrb* mutants PIN3‐GFP did not show a relocation after 6 h gravistimulation, while *hhb* mutants showed PIN3‐GFP persisting at the outer sides of endodermal cells at the bottom hypocotyl side even at later time points (24 h). Scale bar: 60 μm (left‐side image) and 20 μm (gravity stimulated *PIN3::PIN3‐GFP*), and 2 cm for phenotype images. Arrowheads depict outer sides of endodermal cells. Arrow indicates gravity direction.

The cell‐to‐cell auxin flow depends on the pin‐formed (PIN) auxin exporters (Adamowski and Friml, 2015). PINs are plasma membrane (PM)‐based auxin transporters of which the cellular polar localization controls auxin flow and thus asymmetric auxin distribution in various developmental processes, including tropic responses (Wiśniewska *et al*., 2006; Kleine‐Vehn and Friml, 2008b; Kleine‐Vehn *et al*., 2008a). Based on mutant phenotype, expression and localization pattern, as well as polarity change after tropic stimuli, PIN3 appears to be the main mediator of the lateral directional auxin transport during hypocotyl tropic responses (Friml *et al*., 2002; Harrison and Masson, 2008; Ding *et al*., 2011; Rakusová *et al*., 2011, 2016). Following gravistimulation, PIN3 in hypocotyl endodermal cells polarizes to the bottom PMs, which mediates auxin flow to and corresponding auxin accumulation at the lower organ side leading to growth promotion there and hypocotyl bending (Figure 1a,b; Rakusová *et al*., 2011, 2016). At the later stage of gravitropic bending, auxin distribution equalizes and hypocotyl terminates bending (Figure 1a,b; Rakusová *et al*., 2016). This bending termination is triggered by the auxin‐mediated feedback regulation of PIN3 polar localization in endodermal cells at the lower side of the hypocotyls. After the increased auxin accumulation at the lower side of the hypocotyl, PIN3 at the outer PMs is specifically targeted for lytic degradation, whereas PIN3 at the inner cell sides persists (Rakusová *et al*., 2016). Due to the activity of PIN3 at the inner cell sides, the auxin accumulation in the lower epidermis dissipates, asymmetric elongation stops and bending terminates (Rakusová *et al*., 2016). Hence, two independent PIN3 polarization events occur during hypocotyl gravitropism. Firstly, gravity‐induced PIN3 polarization to the lower side of endodermal cells and secondly the auxin‐mediated feedback regulation on PIN3 (termed as inner‐lateralization) restricting PIN3 at the inner sides of endodermal cells (Figure 1a,b; Rakusová *et al*., 2016). If this model is correct, the defects in gravity‐induced PIN3 polarization are expected to correlate with the decreased gravitropic bending of hypocotyl, whereas defects in auxin feedback regulation of PIN3 localization at later stages should lead to the bending termination defect and hyperbending.

Clathrin‐mediated endocytosis and constitutive protein cycling via ARF‐GEF GNOM‐mediated trafficking contribute to intracellular PIN3 polarity regulation (Kleine‐Vehn *et al*., 2010; Naramoto *et al*., 2010; Rakusová *et al*., 2015). Both gravity‐ and auxin‐induced PIN3 relocations require PINOID (PID) and related WAG1 and WAG2 serine/threonine protein kinases activity (Michniewicz *et al*., 2007; Rakusová *et al*., 2011, 2016). Protein degradation and *de novo* protein synthesis do not seem to be crucial parts of the mechanism for gravity‐induced PIN3 relocation (Rakusová *et al*., 2011); however, vacuolar targeting and PIN degradation (Baster *et al*., 2013) contribute to auxin‐induced PIN3 polarization (Rakusová *et al*., 2016). Despite these initial insights into cellular mechanisms of PIN localization and polarization, the molecular mechanism and specific regulators of gravity‐ and auxin feedback‐mediated PIN3 polarization remain elusive.

Here we designed a forward genetic screen to identify unknown regulators of PIN3 polarization and hypocotyl gravitropic response. We identified two groups of mutants: (i) *hypocotyl reduced bending* (*hrb*); and (ii) *hypocotyl hyperbending* (*hhb*) defective in gravity‐induced and auxin feedback‐induced PIN3 polarizations, respectively. Mapping of several mutants and characterization of corresponding genes confirmed that this screen will open multiple avenues into our understanding on mechanisms of PIN polarization and hypocotyl gravitropism.

## Results

### A forward genetic screen for mutants defective in PIN3 polarization during hypocotyl gravitropic bending

To identify novel molecular regulators of PIN3 polarity switches during hypocotyl gravitropic response, we designed a forward genetic screen that would allow us to isolate mutants linking PIN3 polarization defects at the cellular level and their macroscopic manifestation of hypocotyl gravitropic bending (Figure 1c).

An ethyl methylsulfonate (EMS)‐mutagenized *PIN3::PIN3‐GFP* (Col‐0 background) population was established and initially screened for mutants affected in hypocotyl gravitropic bending, and only in the following screening round for defective PIN3‐GFP polarization (Figure 1c). We performed the primary screen of ~66 800 M2 (100 M2 pools representing 2671 M1 families) 3‐day‐old etiolated seedlings based on the hypocotyl hypo‐ or hyperbending following 24 h of gravistimulation. A secondary screen on 127 selected M2 candidates in the M3 generation confirmed the heritable and stable defects in the bending response. Moreover, the PIN3 relocation in the endodermal cells was analyzed in the selected 37 candidates with the strongest phenotypes by means of confocal microscopy 6 h after gravistimulation for mutants with reduced gravitropic bending or 24 h after stimulation for hyperbending mutants (Figure 1c; Table S1). We also tested the effect of externally applied auxin on PIN3‐GFP polarity for both groups of mutants to further refine their classification (Table S1). A detailed analysis of bending response and PIN3‐GFP polarization following gravistimulation as well as auxin‐mediated PIN3‐GFP relocation allowed the classification of the mutants into the two subgroups: (i) mutants with defective bending named *hypocotyl reduced bending* (*hrb*); and (ii) mutants showing an increased hypocotyl bending named *hypocotyl hyperbending* (*hhb*).

To identify the causal mutations for selected mutants, mapping populations were generated by crossing the mutants with the parental line *PIN3::PIN3‐GFP*. F2 seedlings that showed the expected phenotype after gravity stimulation of etiolated hypocotyls were selected based on the same criteria as for the macroscopic primary screen. After that, whole‐genome sequencing [next‐generation sequencing (NGS); see Experimental procedures] was performed on selected mutants to identify the genes that we anticipated to play a role in regulation of directional PIN3‐mediated auxin flow in hypocotyl gravitropism.

### Gravity‐ and auxin‐induced PIN3 polarization defects in *hrb* and *hhb* mutants

**Figure 2:**
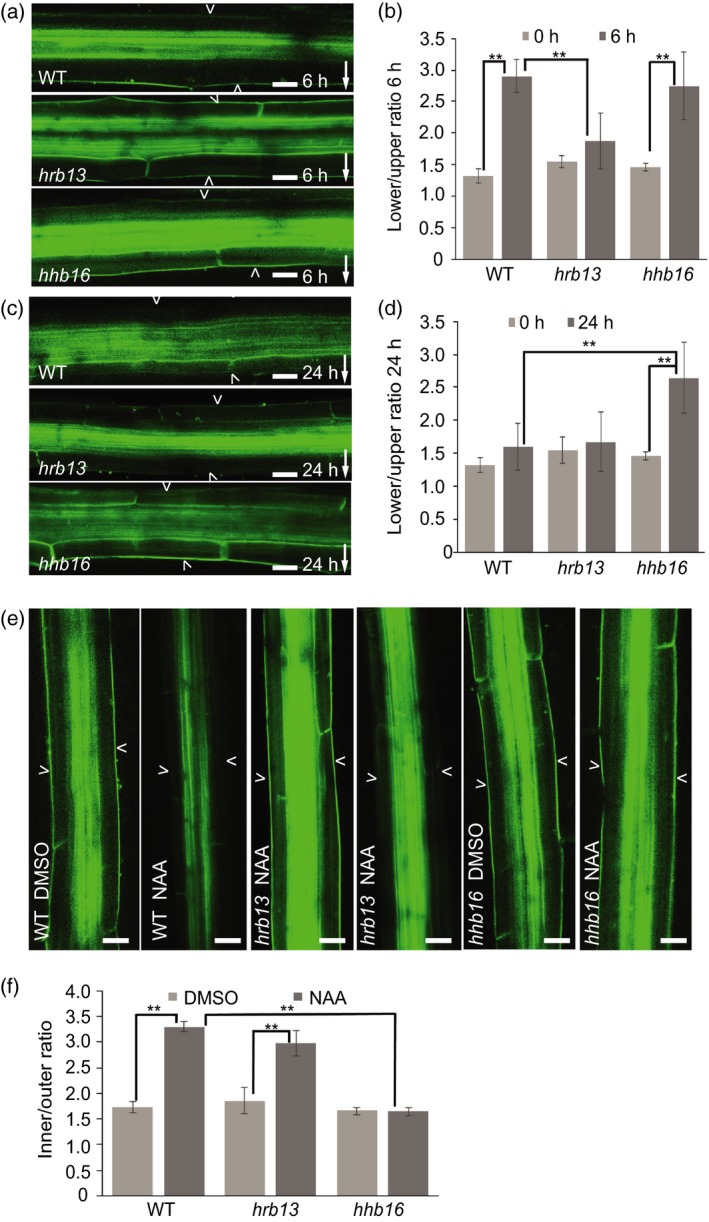
Defective PIN3‐GFP polarization events in *hrb13* and *hhb16* mutants. (a) PIN3‐GFP in wild‐type, *hrb13* mutant, *hhb16* mutant after 6 h gravity stimulation. Scale bar: 20 μm. Arrowheads depict outer sides of endodermal cells. Arrow indicates gravity direction. (b) Quantification of PIN3‐GFP signal in *hrb13* mutant, *hhb16* mutant after 6 h gravistimulation. PIN3‐GFP fluorescence was compared between outer side of endodermal cells at the lower and upper sides of hypocotyl. Error bars are SE (*N *> 15 seedlings for each replicate, Student's *t*‐test, **P < 0.05). (c) PIN3‐GFP in wild‐type, *hrb13* mutant, *hhb16* mutant after 24 h gravity stimulation. Scale bar: 20 μm. Arrowheads depict outer sides of endodermal cells. Arrow indicates gravity direction. (d) Quantification of PIN3‐GFP signal in *hrb13* mutant, *hhb16* mutant after 24 h gravistimulation. PIN3‐GFP fluorescence was compared between outer side of endodermal cells at lower and upper sides of hypocotyl. Error bars are SE (*N *> 15 seedlings for each replicate, Student's *t*‐test, **P < 0.05). (e) PIN3‐GFP in wild‐type, *hrb13* mutant, *hhb16* mutant after 4 h dimethylsulfoxide (DMSO) or NAA treatment. Scale bar: 20 μm. Arrowheads depict outer sides of endodermal cells. (f) Quantification of PIN3‐GFP signal in *hrb13* mutant, *hhb16* mutant after 4 h NAA treatment. PIN3‐GFP fluorescence was compared between inner and outer sides of endodermal cells. Error bars are SE (*N *> 15 seedlings for each replicate, Student's *t*‐test, **P < 0.05).

Initially, the role of endodermal PIN3 in hypocotyl was tested using lines expressing PIN3‐YFP specifically in endodermal cells under *SCARECROW* (*SCR*) promoter. This construct to a large extent rescued hypocotyl gravitropism of *pin3* mutant and a clear PIN3 repolarization was observed following gravistimulation (Rakusová *et al*., 2011). Therefore, we analyzed PIN3 polarization after gravistimulation in the *hrb* and *hhb* mutant groups. All selected mutants belonging to the *hrb* group displayed a defective PIN3‐GFP relocation 6 h after gravistimulation (Figures 2a,b and S2a,b), but showed a normal second PIN3 relocation at the later stage of gravitropic response that is mediated by auxin (Figure 2c,d). On the other hand, *hhb* mutants with a hyperbending phenotype showed defects in the second PIN3 polarization 24 h after gravistimulation (Figures 2c,d and S2c,d), but with a normal gravity‐induced PIN3 polarization 6 h after gravistimulation (Figure 2a,b).

Next, we tested PIN3 polarization in response to externally applied auxin in both *hrb* and *hhb* mutants. First, to avoid interference of the signal in vasculature, we used the *SCR::PIN3‐YFP* line (Figure S3a,b) to confirm the previously reported auxin effect on enrichment of PIN3 at the inner side of endodermal cells (Rakusová *et al*., 2016). As another control, we quantified PIN3‐GFP intensity in vascular tissue of *PIN3::PIN3‐GFP* after auxin (1‐Naphthaleneacetic acid [NAA], 4 h) treatment showing that auxin has no effect on the GFP signal in vasculature under our experimental conditions (Figure S3c). Under these conditions, the *hrb* mutants showed a normal PIN3 inner‐lateralization following auxin treatment (Figures 2e,f and S3d,e); however, auxin‐induced PIN3 inner‐lateralization was severely defective in *hhb* mutants (Figures 2e,f and S3f,g). Thus, the *hrb* mutants had typically specific defects in gravity‐induced PIN3‐GFP polarization, whereas *hhb* mutants were defective in the auxin feedback on PIN3‐GFP at later stages of gravitropic bending and in auxin‐mediated PIN3‐GFP polarization in response to external auxin application.

These specific PIN3 polarization defects in *hrb* and *hhb* mutants confirmed the previous notions that gravity‐induced PIN3 relocation is required for gravitropic bending (Friml *et al*., 2002; Rakusová *et al*., 2011) and the auxin feedback on PIN3 polarity for its termination (Rakusová *et al*., 2016). These results also validated our approach to use macroscopic phenotype in hypocotyl bending to obtain mutants in PIN3 polarization.

### Multiple PIN‐related developmental phenotypes in *hrb* and *hhb* mutants

In addition to gravitropism, PIN3 also plays an important role in apical hook formation (Žádníková *et al*., 2010), lateral root initiation (Marhavý *et al*., 2013), root gravitropism (Harrison and Masson, 2008; Kleine‐Vehn *et al*., 2010) and phototropism (Ding *et al*., 2011). Indeed the selected mutants showed multiple PIN3‐related phenotypes (Table S1). We selected representatives from *hrb* and *hhb* mutant groups to characterize other potentially PIN3‐related phenotypes. After 24 h gravity stimulation, *hrb*2 mutant showed a reduced hypocotyl bending, whereas *hhb*9 mutant showed a hyperbending phenotype (Figure S4a,e). In addition, *hrb*2 and *hhb*9 showed a reduced and a hyperbending phototropic response, respectively (Figure S4b,f). The *hrb*2 mutant not only showed a reduced bending but also showed defects in apical hook development (Figure S4d,g,h). Additionally, the lateral root formation was impaired in *hrb*2 mutant (Figure S4c). All the above‐described phenotype features were tested on all selected 37 candidates in both groups of mutants (Table S1).

Taken together, all these observed phenotypes show that many hypocotyl gravitropism mutants are defective also in other PIN‐related developmental processes, indicating that the respective genes play a broader role in cell polarity or auxin transport.

### Next‐generation sequencing‐based mapping of *hrb17* and *hhb13* mutations

Taking advantage of the NGS technique (see Experimental procedures for details), we obtained a list of candidate genes for several selected mutants. Here, we describe in more detail candidate genes from the two mutants, one from the category of *hypocotyl reduced bending* mutants (*hrb17* mutant) and one with an *hyperbending* hypocotyl mutants (*hhb13* mutant). Within the sequencing list, we screened for mutations in genes potentially playing a role in PIN3 polarization and gravitropic response. The selected candidate genes for mutants *hrb17* and *hhb13* (Table S2) represented good candidates as intracellular dynamics regulators that might be involved in the fine‐tuning of the PIN3 polarity regulation. Based on the plots from the whole‐genome sequencing results, we selected possible intervals with increased mutation abundance for both *hrb17* and *hhb13* mutants (Figure S5a,b, intervals marked with green squares). From these intervals, only mutations with a frequency higher than 70% and only mutations causing amino acid changes, open reading frame shifts or incorporating a STOP codon in the coding sequences were selected and confirmed by resequencing. This strategy allowed to identify the corresponding causal mutations.

### *hrb17* mutant is defective in *SCARECROW* transcription factor

**Figure 3:**
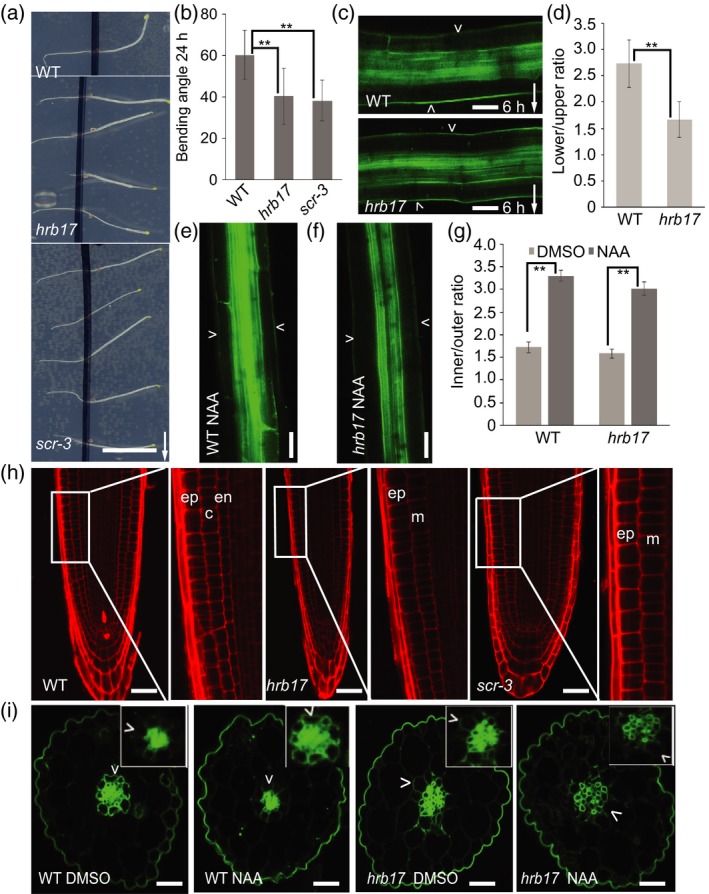
Characterization of *hrb17* mutant. (a) Images of 24 h gravity stimulated *hrb17* mutant and *scr‐3* mutant hypocotyl. Scale bar: 2 cm. Arrow indicates gravity direction. (b) Quantification of hypocotyl bending angle after 24 h gravistimulation in the *hrb17* and *scr‐3* mutants. Error bars are SE (*N *> 20 seedlings for each replicate, Student's *t*‐test, **P < 0.05). (c) PIN3‐GFP in wild‐type and *hrb17* mutant after 6 h of gravistimulation. Scale bar: 20 μm. Arrowheads depict the outer sides of endodermal cells. Arrow indicates gravity direction. (d) Quantification of PIN3‐GFP signal in the *hrb17* mutant after 6 h gravity stimulation. PIN3‐GFP fluorescence was compared between the outer side of endodermal cells at the lower and upper sides of hypocotyl. Error bars are SE (*N *> 15 seedlings for each replicate, Student's t‐test, **P < 0.05). (e,f) PIN3‐GFP in wild‐type and *hrb17* mutant hypocotyls following 4 h of NAA treatment. Scale bar: 20 μm. Arrowheads depict the outer sides of endodermal cells. (g) Quantification of PIN3‐GFP signal following dimethylsulfoxide (DMSO) or NAA treatment in wild‐type and *hrb17* mutant. PIN3‐GFP fluorescence was compared between the inner and outer sides of endodermal cells. Error bars are SE (*N *> 15 seedlings for each replicate, Student's test, **P < 0.05). (h) Root architecture of wild‐type and *hrb17* and *scr‐3* mutants. *hrb17* and *scr‐3* mutants lack endodermal cell layer. Scale bar: 20 μm. Abbreviations: c, cortex; en, endodermis; ep, epidermis; m, irregular mutant cell layer (*N *> 15 seedlings for each replicate). (i) Transverse sections of wild‐type and *hrb17* mutant. Three‐day‐old etiolated seedlings were transferred onto new plates with DMSO or NAA for 4 h, and transversal sections through the hypocotyls were analyzed. Scale bars: 20 μm. Arrowheads depict the outer sides of endodermal cells (*N *> 15 seedlings for each replicate).

Mutant *hrb17* showed a reduced hypocotyl gravitropic bending phenotype (Figure 3a,b), defects in gravity‐induced PIN3 polarization (Figure 3c,d), but a normal auxin‐induced PIN3 relocation (Figure 3e,f,g). The *hrb17* mutant carried mutations in the genes AT3G48170, AT3G54220, AT3G59040 and AT3G61240 (Table S2). The most likely candidate was AT3G54220, namely *SCARECROW* (*SCR*). *scr* mutant had been previously described to show a reduced hypocotyl gravitropic bending phenotype (Fukaki *et al*., 1996, 1998). *scr* mutant displayed a comparable defective gravitropic bending phenotype to that of *hrb17* mutant (Figure 3a,b). Similarly to the *scr* strong allele, *hrb17* frequently lacked an endodermal cell layer in roots and hypocotyls, and instead exhibited a single cell file with mixed cortex/endodermal identity as described for *scr* mutant (Figures 3h and S6a; Fukaki *et al*., 1996, 1998).

To further confirm the *hrb17* mutant is indeed an allele of *scr*, we performed an allelic test. We crossed *scr‐3* with *hrb17* mutant and tested the hypocotyl bending of F1 seedlings after gravity stimulation, F1 seedlings showed a reduced hypocotyl gravitropic bending phenotype comparable to *scr‐3* and *hrb17* mutants, confirming that *hrb17* is a *scr* allele in terms of hypocotyl gravitropism (Figure S6b,c). In addition, under our experimental conditions, both *hrb17* and *scr‐3* mutants along with their F1 progeny also showed defects in root gravitropism (Figure S6d).

The identical phenotypes including hypocotyl gravitropic defect in *scr* and *hrb17* allele confirm that SCR activity is required for hypocotyl gravitropism. This also verifies the viability of our approach for identifying regulators involved in endodermal cell specification is important for hypocotyl gravitropism and gravity‐induced PIN3 polarization.

### SCR‐mediated endodermis specification is required for gravity‐ but not auxin‐induced PIN3 polarization

Next we addressed a possible mechanism underlying the SCR role in hypocotyl gravitropism and PIN3 polarizations. First, we tested whether the PIN3 polarization events during hypocotyl gravitropism require the endodermal cell identity. The observed defect in gravity‐induced PIN3 polarization may be due to defects in sensing gravity or downregulation of *LAZY1* family genes (Taniguchi *et al*., 2017). Gravity sensing by starch sedimentation in endodermal cells is important for gravity response and asymmetric distribution of auxin. It has been reported that *scr* mutant has defects in starch sedimentation in endodermal cells (Fukaki *et al*., 1998). *hrb17* allele had less starch (Figure S7a,b), which would lead to sedimentation defects in endodermal cells. The potential defective starch sedimentation in endodermal cells of both *scr* and *hrb17* mutants can explain the defects in defective hypocotyl gravitropic bending and gravity‐induced PIN3 polarization.

To evaluate the PIN3 localization in distinct hypocotyl cell layers, we analyzed transversal sections of etiolated hypocotyls in the wild‐type *PIN3::PIN3‐GFP* line and in the *hrb17* allele of the *scr* mutant. An apolar PIN3‐GFP localization was detected in endodermal cells of the wild‐type seedlings and in the irregular cells of the *hrb17* mutant (Figure 3i). In both cases, auxin‐induced PIN3 inner‐lateralization (induced by external auxin treatment) was normal (Figure 3i), consistent with our original observations (Figure 3e,f,g). Next, we tested the cell type specificity of the auxin impact on the PIN3 localization. The PIN3‐GFP signal is generally weaker in cortex cells than in endodermal cells of the wild‐type hypocotyls (Figure S8a,b). To avoid the PIN3‐GFP signal from the neighboring endodermal cells, only the outer lateral cortical cell side was measured and compared following dimethylsulfoxide (DMSO) or NAA treatment. The fluorescence intensity of PIN3‐GFP at the outer cortical cell side did not decrease after NAA treatment (Figure S8a,b). This observation shows that auxin‐mediated PIN3 polarity regulation is more specific to the endodermal cells. Nonetheless, cells with mixed identity in *hrb17* mutant still allow for auxin‐mediated (Figure 3e,f,g,i) but not for gravity‐induced PIN3 polarization (Figure 3c,d).

To address, if endodermal cell identity is important for gravity‐induced PIN3 polarization, we investigated PIN3 polarizations in *endodermal‐amyloplast less 1* (*eal1*) mutant, an allele of SHORT ROOT (SHR) but with an identical reduced shoot gravitropic bending phenotype and endodermal cell defects to *scr* mutant (Fukaki *et al*., 1998; Morita *et al*., 2007). Indeed, *eal1*/*shr* mutant showed the same defects in gravity‐induced PIN3 polarization (Figure S9a,b,d), but a normal auxin‐induced PIN3 polarization (Figures S9c,e and S10a,b); both features similar as observed in *hrb17* mutant. This supports the conclusion that SCR/SHR‐mediated acquisition of endodermal cell identity is required for gravity‐induced but not for auxin‐induced PIN3 polarization.

### *hhb13* mutant is defective in ACTIN2

**Figure 4:**
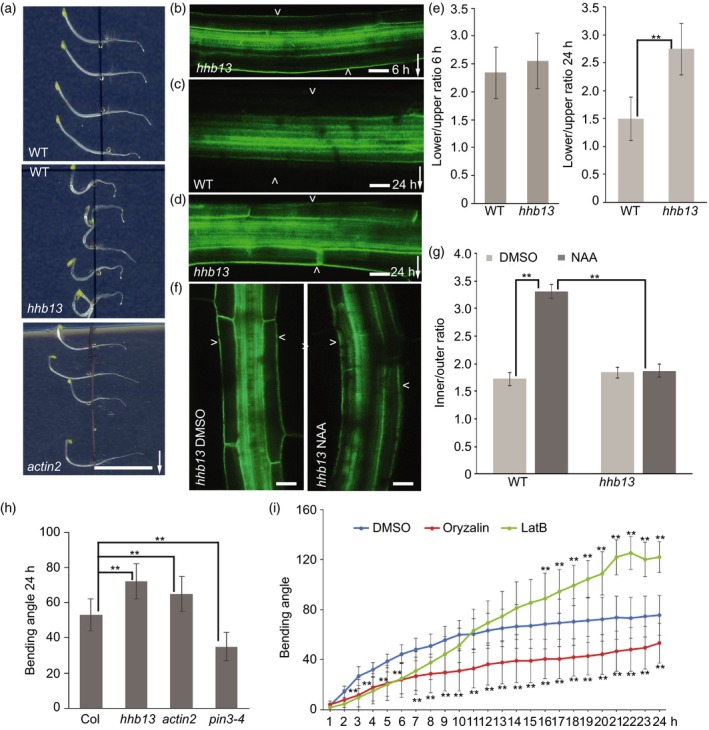
Characterization of *hhb13* mutant. (a) Images of 24 h gravity stimulated wild‐type, *hhb13* and *actin2* mutants. Scale bar: 2 cm. Arrow indicates gravity direction. (b) PIN3‐GFP in *hhb13* mutant after 6 h gravity stimulation. Scale bar: 20 μm. (c) PIN3‐GFP in wild‐type after 24 h gravity stimulation. Scale bar: 20 μm. (d) PIN3‐GFP in *hhb13* mutant after 24 h gravity stimulation. Scale bar: 20 μm. Arrowheads depict the outer sides of endodermal cells. Arrow indicates gravity direction. (e) Quantification of PIN3‐GFP signal after 6 and 24 h gravity stimulation in wild‐type and *hhb13* mutant. PIN3‐GFP fluorescence was compared between the outer side of endodermal cells at the lower and upper sides of hypocotyl. Error bars are SE (*N *> 15 seedlings for each replicate, Student's *t*‐test, **P < 0.05). (f) PIN3‐GFP in *hhb13* mutant after 4 h DMSO or NAA treatment. Scale bar: 20 μm. Arrowheads depict the outer sides of endodermal cells. (g) Quantification of PIN3‐GFP signal after DMSO or NAA treatment in *hhb13* mutant. PIN3‐GFP fluorescence was compared between the inner and outer sides of endodermal cells. Error bars are SE (*N *> 15 seedlings for each replicate, Student's test, **P < 0.05). (h) Bending angle of wild‐type, *hhb13*,* actin2*,* pin3‐4* mutant after 24 h gravistimulation. Error bars are SE (*N *> 25 seedlings for each replicate, Student's *t*‐test, **P < 0.05). (i) Bending kinetics of wild‐type seedlings treated with DMSO, latrunculin B (LatB) and oryzalin. Pictures were taken at 1‐h intervals. Error bars are SE (*N *> 25 seedlings for each replicate, Student's *t*‐test between DMSO and LatB treatment, **P < 0.05; Student's *t*‐test between DMSO and oryzalin treatment, **P < 0.05).

Mutant *hhb13*, displaying a hyperbending hypocotyl phenotype (Figure 4a,h), showed a normal gravity‐induced PIN3 relocation (Figure 4b,e), but with a specific defect in auxin‐induced PIN3 polarization (Figure 4c–g). The *hhb13* mutant carried a mutation in gene AT3G18780, coding for the ACTIN2 (ACT2) protein. An independent mutant allele in ACTIN2 from the T‐DNA SALK collection (SALK_048987; Nishimura *et al*., 2003) showed phenotypes very comparable to the *hhb13* mutant including similar hypocotyl hyperbending response (Figure 4a,h). *ACTIN2* is uniformly expressed in young seedlings and co‐expressed in hypocotyls with *ACTIN7* and *ACTIN8* (McDowell *et al*., 1996a,1996b). To test the contribution of other ACTINs to the hypocotyl gravitropic response, the hypocotyl bending assay was performed in single *actin7* and *actin8* mutants, and higher‐order of *actin* mutants. *actin7* and *actin8* showed normal gravitropic bending after 24 h gravity stimulation, but a higher order of *actin2*,* actin7* and *actin8* mutants showed a hyperbending phenotype (Figure S11). This suggests a more specific role of ACTIN2 in the hypocotyl gravitropic response, but ACTIN7 and ACTIN8 also contribute to the hypocotyl gravitropism.

To further confirm the role of ACTIN2 in regulation of PIN3 polarization, we crossed *actin2* mutant with *PIN3::PIN3‐GFP*. As shown in *hhb13* mutant, *actin2* mutant also showed a normal gravity‐induced PIN3 polarization (Figure S12a,b,c,f), but showed auxin‐induced PIN3 polarization defects at a later stage of gravitropic response (Figure S12d,e,g). Furthermore, externally applied auxin was not able to induce PIN3 polarization into the inner side of endodermal cells in *actin2* mutant (Figure S13a,b). All these data support that ACTIN2 is required for auxin‐induced but not for gravity‐induced PIN3 polarization.

The identical phenotypes including hypocotyl hyperbending and PIN3 polarization in *actin2* and *hhb13* alleles reveal the role of ACTIN in hypocotyl gravitropism. This also verifies the viability of our approach for identifying regulators of auxin‐induced PIN3 polarization based on hypocotyl hyperbending.

### Distinct roles of actin and microtubule cytoskeleton in PIN3 polarization events during gravitropic response

The identification of *actin2* mutant with a hyperbending phenotype (Figure 4a,h) prompted us to test the role of actin and microtubule (MT) cytoskeleton in hypocotyl gravitropism and PIN3 polarization. We used oryzalin, a substance disrupting MTs, and latrunculin B (LatB) targeting actin filaments. Both drugs altered hypocotyl gravity responses (Figure 4i). Oryzalin slowed down hypocotyl bending, whereas LatB initially slowed down but after a prolonged time caused a hypocotyl hyperbending phenotype similar to the *act2* mutant (Figure 4i). However, LatB and oryzalin both inhibited hypocotyl growth under our experimental conditions (Figure S14), but they showed different effects on gravitropism (Figure 4i).

Next, we tested the involvement of actin cytoskeleton and MTs in regulation of PIN3 polarization. We transferred 3‐day‐old dark‐grown seedlings on to 30 μm LatB, 30 μm oryzalin or the same amount of DMSO plates for 1 h, then gravistimulated for 6 or 24 h. Oryzalin inhibited gravity‐induced PIN3 relocation (Figure S15a,b), consistent with its inhibitory effect on hypocotyl bending (Figure 4i). Gravity‐induced PIN3 relocation was also slightly reduced by disrupting the actin cytoskeleton (Figure S16a,b,e). Notably, at the later stage of gravitropic response, LatB inhibited the second PIN3 polarization event to the inner side of endodermal cells, which correlated with and explained the hypocotyl hyperbending response (Figure S16c,d,f).

**Figure 5:**
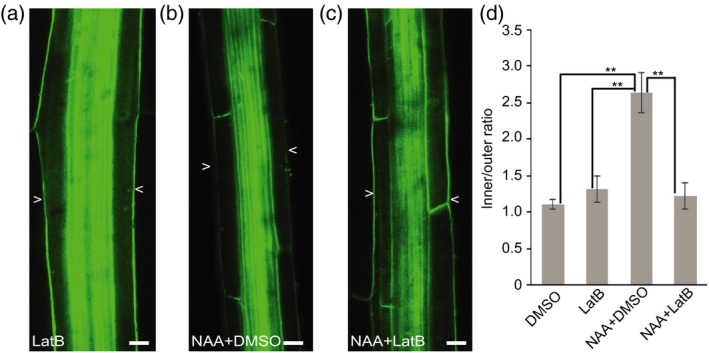
Latrunculin B (LatB) inhibits auxin‐induced PIN3‐GFP polarization. (a) PIN3‐GFP in wild‐type after 30 μm LatB treatment for 4 h. Scale bars: 20 μm. (b) PIN3‐GFP in wild‐type after 10 μm NAA and dimethylsulfoxide (DMSO) co‐treatment for 4 h. Scale bars: 20 μm. (c) PIN3‐GFP in wild‐type after 30 μm LatB and 10 μm NAA co‐treatment for 4 h. Scale bars: 20 μm. Arrowheads depict the outer sides of endodermal cells. (d) Quantification of PIN3‐GFP signal. PIN3‐GFP fluorescence was compared between the inner and outer sides of endodermal cells. Error bars are SE (*N *> 15 seedlings for each replicate, Student's *t*‐test, **P < 0.05).

Therefore, we also tested if LatB can inhibit auxin feedback on PIN3 relocation. We transferred seedlings to the plates supplemented with 30 μm LatB or DMSO for 1 h, then co‐treated with 10 μm NAA for 4 h. Consistent with the effect of LatB on the second PIN3 polarization event, LatB also inhibited auxin‐induced PIN3 relocation to the inner side of endodermal cells (Figure 5b,c,d), but LatB itself has no pronounced effects on PIN3 localization (Figure 5a,d).

Thus, it appears that MTs are required for gravity‐induced PIN3 polarization, whereas actin is more specifically needed for auxin‐mediated PIN3 polarization. Taken together, these observations revealed distinct roles of actin and MT cytoskeletons in PIN polarization events during hypocotyl gravitropism.

## Discussion

### Forward genetic screen for regulators of PIN polarization events during gravitropism

Endogenous and external signals can modify cellular PIN polarity in a plethora of developmental contexts to redirect intercellular auxin fluxes and contribute to the flexible, adaptive plant development (Paciorek and Friml, 2006; Adamowski and Friml, 2015). To identify unknown molecular regulators of these processes, we designed a forward genetic screen based on altered bending of *Arabidopsis thaliana* gravistimulated hypocotyl. Hypocotyl gravitropism involves both gravity‐induced relocation of PIN3 in endodermal cells leading to auxin accumulation at lower side of the organ, followed by auxin‐mediated feedback on PIN3 repolarization equalizing the auxin levels and terminating the bending (Rakusová *et al*., 2011, 2016). Thus, easily scoreable, macroscopic phenotype of hypocotyl bending allowed to successfully identify mutants defective in gravity‐ and auxin‐induced PIN3 polarization. Notably, mutants with *reduced hypocotyl bending* (*hrb*) showed typically defects in gravity‐induced PIN3 polarization, whereas *hypocotyl hyperbending* (*hhb*) mutants had defects in a second, auxin‐mediated polarization event. This confirmed a previous notion (Rakusová *et al*., 2011, 2016) that gravity‐induced PIN3 polarization is required for initiation of the bending and auxin‐mediated PIN3 polarization for the bending response termination.

A number of identified mutants also displayed defects in other auxin transport‐related developmental processes, such as root growth, lateral root development and apical hook development, indicating that the respective genes play a broader role in auxin transport or PIN polarity regulation. Using a whole‐genome NGS‐based technique, we identified several candidates from both *hrb* and *hhb* group mutants as potential novel components in regulation of PIN3 polarization during gravitropism. Among the candidates, *SCARECROW* from *hrb* group and *ACTIN2* from *hhb* group represented two good candidates that were characterized in more detail to confirm that the presented screen is instrumental to identify molecular components of PIN polarization during plant adaptive development.

### SCR‐mediated endodermis specification is required for gravity‐induced PIN3 polarization

One of the *hrb* mutants, *hrb17* mutant, carried a mutation in AT3G54220, the *SCARECROW* gene. Allelic test confirmed that *hrb17* is an allele of *scr* mutant with a reduced hypocotyl gravitropic bending phenotype (Fukaki *et al*., 1996, 1998). The lack of endodermal cells in *scr* mutant alleles raised the question whether PIN3 relocation events require endodermis specification. Confocal laser‐scanning microscopy‐based analysis revealed that *scr*/*hrb17* and *eal1*/*shr* mutants do not show gravity‐induced PIN3 polarization but still showed normal auxin‐mediated PIN3 polarization. This suggests that endodermis cell fate is required specifically for gravity‐ but not auxin‐mediated PIN polarization. This can be explained by defective gravity perception reported for the *scr* mutant, possibly due to diminished number of starch‐containing, gravity‐sensing amyloplasts in both *scr* (Fukaki *et al*., 1998) and *hrb17* mutant alleles. It has been reported that mutations in *LAZY* gene affect early gravity signal transduction and auxin distribution, resulting in altered hypocotyl gravitropic response (Taniguchi *et al*., 2017). Moreover, *LAZY* gene expression is downregulated in the *scr* mutant (Taniguchi *et al*., 2017), therefore it is possible that altered *LAZY* expression in both *scr* and *hrb17* mutant could also be a basis for the altered hypocotyl gravitropic response. However, the precise mechanism how PIN3 polarization is regulated by the SCR‐mediated endodermis cell fate or LAZY1 proteins requires more future insights; nonetheless, characterization of *hrb17* was confirmed as important in endodermis specification in shoot gravitropic response and PIN polarization.

### Distinct roles of actin and microtubules in PIN3 polarization events during gravitropism

The hyperbending phenotype of *hhb13* mutant correlated with a mutation in *ACTIN2* gene and another *actin2* allele showed a comparable hyperbending phenotype similar to *hhb13* mutant as well as the PIN3 polarization defects. The pharmacological interference with actin cytoskeleton also resulted in a hypocotyl hyperbending similar to *actin2* and *hhb13* mutants collectively revealing a role of actin cytoskeleton in gravitropic bending termination. Actin has been suggested as a negative regulator of gravitropism, possibly by preventing a too fast sedimentation of statoliths (Yamamoto and Kiss, 2002; Nakamura *et al*., 2011), but our data suggest a different mode of action. Gravity‐induced PIN3 relocation was not affected in both *hhb13* and *actin2* mutants or following pharmacological interference with actin. In contrast, similar experiments revealed that auxin‐induced PIN3 relocation requires intact actin cytoskeleton. Thus, interference with actin prevents second, auxin‐mediated PIN3 polarization and leads to hypocotyl hyperbending further confirming that auxin feedback on PIN3 polarity mediates termination of the bending response. The single *actin7* and *actin8* mutants showed a normal gravitropic response, but a higher order of *actin* mutants show increasingly more severe gravitropic hypocotyl hyperbending. Thus, despite proposed differential roles of actins in root gravitropism ascribed to the variation in regulation and the diversity in actin sequences (Kandasamy *et al*., 2009), they all act redundantly, albeit with different importance, in termination of hypocotyl gravitropic bending.

We also examined the role of MTs in hypocotyl gravitropism and PIN polarization. Our observations show that MTs are required for both gravity‐induced PIN3 relocation and hypocotyl bending. It remains unclear how MT cytoskeleton may regulate PIN polar trafficking (Geldner *et al*., 2001). It also may be an indirect effect of tissue polarity (Boutté *et al*., 2006) or cell wall defects (Feraru *et al*., 2011).

Generally, the interpretation of involvement of actin and MTs in regulation of PIN3 polarity is complicated due to the possible roles of actin in gravity perception (Yamamoto and Kiss, 2002; Keuskamp *et al*., 2010; Nakamura *et al*., 2011). Also, many different cellular processes such as PIN trafficking, recycling and cell elongation require MTs and actin cytoskeleton (Geldner *et al*., 2001; Friml *et al*., 2002; Kleine‐Vehn *et al*., 2008a; Nick *et al*., 2009; Ambrose *et al*., 2013; Chen *et al*., 2014), making an interpretation of cytoskeletal roles even more difficult. On the other hand, myosin cytoskeleton protein plays different roles in gravitropism response with either affecting amyloplasts sedimentation (Talts *et al*., 2016) or acting as bending sensors in the fiber cells (Okamoto *et al*., 2015). Furthermore, *myosin* loss‐of‐function mutant also affects PIN polarity (Abu‐Abied *et al*., 2018), indicating a more complicated regulation of PIN polarization by actin–myosin complex. Nonetheless, our studies revealed that MTs and actin cytoskeleton have distinct roles in regulation of PIN3 polarization and hypocotyl gravitropic response. Actin would play a more specific role in auxin‐induced PIN3 polarization to terminate bending response, whereas MTs play a more important role for gravity‐induced PIN3 relocation to initiate bending.

Overall, our hypocotyl gravitropism forward genetic screen proved to be a successful strategy to reveal so far uncharacterized regulators of PIN3 polarization during gravitropism. We obtained a collection of *hrb* and *hhb* mutants with defective PIN3 polarization in response to gravity and auxin, respectively. These presented fully molecularly characterized candidates, and the remaining selected mutants provide new insights into PIN polarity regulation in response to both endogenous and external signals during adaptive development.

## Experimental procedures

### Plant material

The following transgenic and mutant lines were used: Columbia (Col‐0), *PIN3::PIN3‐GFP* (Žádníková *et al*., 2010), *scr‐3* (CS3997; Fukaki *et al*., 1996), *actin2* (SALK_048987; Nishimura *et al*., 2003), *actin7* (SALK_131610), *actin8* (GABI_480B07), *endodermal‐amyloplast less 1* (*eal1*; Morita *et al*., 2007) and *SCR::PIN3‐YFP* (Rakusová *et al*., 2011). Mutant combinations with *PIN3::PIN3‐GFP* were generated through genetic crosses.

### Growth conditions

Seeds were sown on plates with half‐strength Murashige and Skoog (½ MS) medium with 1% sucrose agar and stratified at 4°C for 2 days. Germination was induced by exposing plates to light for 24 h before transfer to darkness and cultivation at 21°C for 3 days. Light sources used were Philips GreenPower LED production modules combined with deep red (660 nm)/far red (720 nm)/blue (455 nm), with a photon density of about 140 μmol m^−2 ^sec^−1^. For gravitropic stimulations, plates with 3‐day‐old seedlings were turned 90°. For confocal microscopy, a Zeiss confocal scanning microscope (Zeiss; http://www.zeiss.com) was used. To monitor gravitropic responses, plates were scanned at the indicated time points after gravistimulation. Images were processed in Adobe Photoshop CS. Each experiment was performed at least three times. Bending angles and fluorescence intensities were measured by ImageJ (NIH; http://rsb.info.nih.gov/ij). The fluorescence intensity of PIN3‐GFP was measured as described after gravity stimulation or auxin treatment (Rakusová *et al*., 2011, 2016). PIN3‐GFP fluorescence intensity in hypocotyl transverse sections was measured at the outer and inner cell side of endodermal cell in wild‐type and in *hrb17* mutant using ImageJ. Two replicates of at least 15 seedlings with synchronized germination were processed.

### Phenotype analysis of selected mutants

For gravitropic and phototropic response, 3‐day‐old etiolated seedlings were either gravistimulated for 24 h or illuminated by unilateral light for 24 h. Plates were scanned and bending angle was measured using ImageJ. Hypocotyl length was measured for all mutants in the dark for 4 days. Apical hook was scanned at 24, 30 and 72 h of dark‐grown seedlings after germination. Root phenotype analysis was performed as follows: all the mutants were germinated on solid medium and grown for 3 days, then seedlings were transferred onto new plates with 100 nm NAA, DMSO and grown for another 3 or 7 days.

### Quantification of PIN3‐GFP/YFP intensity in hypocotyl

All measurements were performed using ImageJ software. For the quantification of PIN3‐GFP/YFP polarization, the intensity of PIN3‐GFP/YFP at endodermal cells was measured (Figure S1a,b). After gravity stimulation, the ratio was calculated between the outer side of endodermal cells at lower and the upper side of horizontally placed hypocotyls (marked with a blue line, Figure S1b; Rakusová *et al*., 2011). For auxin and co‐treatment with other chemicals, the ratio was calculated between the inner side (marked with pink line) and outer side (marked with blue line) of endodermal cells (Figure S1a; Rakusová *et al*., 2016). To confirm the specificity of the PIN3 behavior in endodermal cells following auxin treatment, PIN3‐GFP signals in vasculature and cortex cells were also evaluated. The GFP signal in vasculature was selected (as highlighted white square area in Figure S1a) and calculated as a mean of the fluorescence signal per vasculature/hypocotyl after DMSO or auxin treatment. To measure the PIN3‐GFP intensity changes in cortex cells, only the outer cortex cell side was measured (marked with red line in Figure S1a) after DMSO or auxin application. As an additional control for the accuracy of the *PIN3::PIN3‐GFP* polarity measurements, the *SCR::PIN3‐YFP* line was used, when PIN3 is expressed only in endodermal cells and thus confirmed the same behavior of PIN3 protein upon the gravitropism as well as after auxin treatment. In all cases, at least 20 hypocotyls were measured and the ratio was calculated from the mean.

### Quantification of hypocotyl growth

Three‐days etiolated Col‐0 seedlings were transferred onto new plates supplied with either DMSO or 2, 5, 10 and 30 μm of LatB and oryzalin. All the plates were kept growing in the dark for 24 h. After 24 h, hypocotyl length was measured using ImageJ. Three biological replicates of more than 25 seedlings were used with the same significant results.

### Apical hook development

Seeds were put on ½ MS medium and germination was induced under light for 16 h, then plates were covered with aluminum foil. Apical hook development was recorded at indicated time points. Angles between the hypocotyl axis and cotyledons were measured by ImageJ as described previously (Žádníková *et al*., 2010).

### Pharmacological treatments

Wild‐type seedlings were germinated and grown vertically on ½ MS with 1% sucrose agar plates at 21°C for 3 days. Treatments in the dark were done by transfer and incubation of 3‐day‐old etiolated seedlings on solid medium supplemented with NAA (10 μm; DUCHEFA BIOCHEMIE, Haarlem, The Netherlands), LatB (30 μm; Sigma‐Aldrich Handels GmbH, Vienna, Austria) and oryzalin (30 μm; Duchefa). All co‐treatments with NAA were done after 1 h of pre‐treatment with the drug followed by 4 h of co‐treatment with NAA, 6 or 24 h gravity stimulation. Control treatments contained an equivalent amount of solvent (DMSO; Sigma‐Aldrich). For all comparisons, at least three independent experiments were carried out, giving the same significant results.

### Real‐time bending kinetic analysis

Gravity response of seedlings was recorded at 1‐h intervals for 48 h at 21°C with an infrared light source (880 nm LED; Velleman, Belgium) by a spectrum‐enhanced camera (EOS035 Canon Rebel Xti, 400DH) with built‐in clear wideband‐multicoated filter and standard accessories (Canon Belgium NV/SA, Machelen, Diegem, Belgium) and operated by the EOS utility software. Angles of hypocotyls were measured by ImageJ (National Institutes of Health, http://rsb.info.nih.gov/ij). A minimum of 20 seedlings with synchronized germination start were processed.

### Ethyl methylsulfonate mutagenesis of Arabidopsis seeds and mutant forward genetic screen

M2 seedlings, progenies of 2671 M1 0.3% EMS‐mutagenized Arabidopsis *PIN3::PIN3‐GFP* (ecotype Columbia‐0) plants were analyzed by scoring the gravity bending response of hypocotyls. M3 selfed progenies were rescreened with the same criteria and observed under a confocal microscope for abnormal intracellular localizations of the PIN3‐GFP signal in hypocotyls.

### Starch staining

Seedlings were grown vertically in darkness for 3 days after germination on ½ MS medium with 1% sucrose agar. All the seedlings were fixed in FAA solution [10% formaldehyde (v/v), 5% acetic acid (v/v) and 50% ethanol (v/v)], as previously described (Fukaki *et al*., 1998). After fixation, seedlings were washed in 70% ethanol three times, and stained with Lugol solution (Sigma‐Aldrich) for 3 min. Seedlings were mounted on slides with clearing solution [chloral hydrate:glycerol:water (8:1:2, W:V:V)] for 2 h at normal temperature. Samples were observed using OLYMPUS BX53 microscopy.

### Whole‐genome sequencing

The identified mutants that displayed an altered gravitropic bending response and a defective PIN3‐GFP relocation in the endodermal cells of hypocotyls were back‐crossed into *PIN3::PIN3‐GFP*. The seedlings for the whole‐genome sequencing were selected from a F2‐segregating population. DNA from 60–80 seedlings was isolated using membrane binding DNeasy Plant kit from Qiagen, and sent for whole‐genome sequencing (BGI, http://www.genomics.cn/en/index). The candidate genes with point mutations that introduced a STOP codon or caused amino acid changes were resequenced for confirmation. The list of candidate genes for *hrb17* and *hhb13* is presented in Table S2.

### Transverse sections

Four‐day‐old etiolated seedlings were fixed for 1 h in 4% paraformaldehyde (Serva) in MTSB (50 mm PIPES, 5 nm EGTA, 1 mm MgSO4, pH 6.8) and immobilized in 5% (w/v) water solution of low‐melting agarose (Sigma‐Aldrich). Agarose blocks were mounted onto a Motorized Advance Vibroslice and 100‐μm transversal sections through the hypocotyls were observed with a Zeiss 700 confocal microscope.

### Propidium iodide staining of hypocotyl

Three‐days etiolated seedlings were fixed in FAA solution overnight at 4°C, then seedlings were incubated with propidium iodide (PI; 20 μg ml^−1^) for 20 min. After staining, seedlings were washed with H_2_O twice. Seedlings were mounted on slides with clearing solution, and the upper parts of the hypocotyls were observed with a Zeiss 700 confocal microscope. For roots, 3‐days light‐grown seedlings were stained with PI (20 μg ml^−1^) for 5 min, and seedlings were mounted on slides with water and observed with a Zeiss 800 confocal microscope.

### Statistical analysis

All statistical analysis was performed using Student's *t*‐test in excel (Microsoft 2010) with a significant difference (*P *< 0.05).

## Funding

This work was supported by the European Research Council (project ERC‐2011‐StG‐20101109‐PSDP) and European Social Fund (CZ.1.07/2.3.00/20.0043) to JF. HR was supported by the Agency for Innovation by Science and Technology (IWT) predoctoral fellowship. HH was supported by China Scholarship Council (CSC scholarship).

## Author contributions

HR, HH and JF designed the experiments; HR, HH and PV conducted the experiments and analyzed data; HR, HH and JF wrote the manuscript.

## Competing financial interest

The authors declare no competing financial interests.

## Supporting information


**Figure S1.** Schemes representing cellular membranes used for PIN3‐GFP/YFP quantification.

**Figure S2.** Gravity‐induced PIN3‐GFP relocation in *hrb* and *hhb* mutants.

**Figure S3.** Auxin‐induced PIN3‐GFP inner‐lateralization in *hrb* and *hhb* mutants.

**Figure S4.** Multiple PIN‐related phenotype in the *hrb* and *hhb* mutants.

**Figure S5.** Plots of mutation frequency in the genome of *hrb17* and *hhb13* mutants.

**Figure S6.** Allelic test of *hrb17* mutant.

**Figure S7.** Starch staining in wild‐type and *hrb17* mutant.

**Figure S8.** Auxin‐induced PIN3‐GFP relocation in cortical cell.

**Figure S9.** Gravity‐induced PIN3 polarization in *eal1*/*shr* mutant.

**Figure S10.** Auxin‐induced PIN3 polarization in *eal1*/*shr* mutant.

**Figure S11.** Bending angle of Col, *actin2*,* actin7* and *actin8*, and higher‐order *actin* mutants.

**Figure S12.** Gravity‐induced PIN3 polarization in *actin2* mutant.

**Figure S13.** Auxin‐induced PIN3 polarization in *actin2* mutant.

**Figure S14.** LatB and oryzalin inhibit hypocotyl growth.

**Figure S15.** Oryzalin inhibits gravity‐induced PIN3 relocation.

**Figure S16.** LatB does not affect gravity‐induced PIN3 relocation.

**Table S1.** Phenotype analysis of 37 candidate mutants from the forward genetic screen.

**Table S2.** List of candidate genes for *hrb17* and *hhb13* mutants.

## References

[tpj14301-bib-0001] Abu‐Abied, M. , Belausov, E. , Hagay, S. , Peremyslov, V. , Dolja, V. and Sadot, E. (2018) Myosin XI‐K is involved in root organogenesis, polar auxin transport, and cell division. J. Exp. Bot. 69, 2869–2881. 10.1093/jxb/ery112. 29579267 PMC5972647

[tpj14301-bib-0002] Adamowski, M. and Friml, J. (2015) PIN‐dependent auxin transport: action, regulation, and evolution. Plant Cell, 27, 20–32. 10.1105/tpc.114.134874. 25604445 PMC4330589

[tpj14301-bib-0003] Ambrose, C. , Ruan, Y. , Gardiner, J. , Tamblyn, L.M. , Catching, A. , Kirik, V. , Marc, J. , Overall, R. and Wasteneys, G.O. (2013) CLASP interacts with sorting nexin 1 to link microtubules and auxin transport via PIN2 recycling in *Arabidopsis thaliana* . Dev. Cell, 24, 649–659. 10.1016/j.devcel.2013.02.007. 23477787

[tpj14301-bib-0004] Baster, P. , Robert, S. , Kleine‐Vehn, J. , Vanneste, S. , Kania, U. , Grunewald, W. , De Rybel, B. , Beeckman, T. and Friml, J. (2013) SCF^TIR1/AFB^‐auxin signalling regulates PIN vacuolar trafficking and auxin fluxes during root gravitropism. EMBO J. 32, 260–274. 10.1038/emboj.2012.310. 23211744 PMC3553380

[tpj14301-bib-0005] Boutté, Y. , Crosnier, M.T. , Carraro, N. , Traas, J. and Satiat‐Jeunemaitre, B. (2006) The plasma membrane recycling pathway and cell polarity in plants: studies on PIN proteins. J. Cell Sci. 119, 1255–1265. 10.1242/jcs.02847. 16522683

[tpj14301-bib-0006] Chen, X. , Grandont, L. , Li, H. , Hauschild, R. , Paque, S. , Abuzeineh, A. , Rakusova, H. , Benkova, E. , Perrot‐Rechenmann, C. and Friml, J. (2014) Inhibition of cell expansion by rapid ABP1‐mediated auxin effect on microtubules. Nature, 516, 90–93. 10.1038/nature13889. 25409144 PMC4257754

[tpj14301-bib-0007] Ding, Z. , Galvan‐Ampudia, C.S. , Demarsy, E. ***et al.*** (2011) Light‐mediated polarization of the PIN3 auxin transporter for the phototropic response in *Arabidopsis* . Nat. Cell Biol. 13, 447–452. 10.1038/ncb2208. 21394084

[tpj14301-bib-0008] Feraru, E. , Feraru, M.I. , Kleine‐Vehn, J. , Martinière, A. , Mouille, G. , Vanneste, S. , Vernhettes, S. , Runions, J. and Friml, J. (2011) PIN polarity maintenance by the cell wall in *Arabidopsis* . Curr. Biol. 21, 338–343. 10.1016/j.cub.2011.01.036. 21315597

[tpj14301-bib-0009] Friml, J. , Wiśniewska, J. , Benková, E. , Mendgen, K. and Palme, K. (2002) Lateral relocation of auxin efflux regulator PIN3 mediates tropism in *Arabidopsis* . Nature, 415, 806–809. 10.1038/415806a. 11845211

[tpj14301-bib-0010] Fukaki, H. , Fujisawa, H. and Tasaka, M. (1996) SGR1, SGR2, SGR3: novel genetic loci involved in shoot gravitropism in *Arabidopsis thaliana* . Plant Physiol. 110, 945–955. 10.1104/pp.110.3.945. 8819871 PMC157794

[tpj14301-bib-0011] Fukaki, H. , Wysocka‐Diller, J. , Kato, T. , Fujisawa, H. , Benfey, P.N. and Tasaka, M. (1998) Genetic evidence that the endodermis is essential for shoot gravitropism in *Arabidopsis thaliana* . Plant J. 14, 425–430. 10.1046/j.1365-313X.1998.00137.x. 9670559

[tpj14301-bib-0012] Geldner, N. , Friml, J. , Stierhof, Y.D. , Jürgens, G. and Palme, K. (2001) Auxin transport inhibitors block PIN1 cycling and vesicle trafficking. Nature, 413, 425–428. 10.1038/35096571. 11574889

[tpj14301-bib-0013] Harmer, S.L. and Brooks, C.J. (2018) Growth‐mediated plant movements: hidden in plain sight. Curr. Opin. Plant Biol. 41, 89–94. 10.1016/j.pbi.2017.10.003. 29107827 PMC5826749

[tpj14301-bib-0014] Harrison, B.R. and Masson, P.H. (2008) ARL2, ARG1 and PIN3 define a gravity signal transduction pathway in root statocytes. Plant J. 53, 380–392. 10.1111/j.1365-313X.2007.03351.x. 18047472

[tpj14301-bib-0015] Kandasamy, M.K. , McKinney, E.C. and Meagher, R.B. (2009) A single vegetative actin isovariant overexpressed under the control of multiple regulatory sequences is sufficient for normal *Arabidopsis* development. Plant Cell, 21, 701–718. 10.1105/tpc.108.061960. 19304937 PMC2671709

[tpj14301-bib-0016] Keuskamp, D.H. , Pollmann, S. , Voesenek, L.A. , Peeters, A.J. and Pierik, R. (2010) Auxin transport through PIN‐FORMED 3 (PIN3) controls shade avoidance and fitness during competition. Proc. Natl Acad. Sci. USA 107, 22 740–22 744. 10.1073/pnas.1013457108. 21149713 PMC3012496

[tpj14301-bib-0017] Kleine‐Vehn, J. and Friml, J. (2008b) Polar targeting and endocytic recycling in auxin‐dependent plant development. Annu. Rev. Cell Dev. Biol. 24, 447–473. 10.1146/annurev.cellbio.24.110707.175254. 18837671

[tpj14301-bib-0018] Kleine‐Vehn, J. , Langowski, L. , Wiśniewska, J. , Dhonukshe, P. , Brewer, P.B. and Friml, J. (2008a) Cellular and molecular requirements for polar PIN targeting and transcytosis in plants. Mol. Plant, 1, 1056–1066. 10.1093/mp/ssn062. 19825603

[tpj14301-bib-0019] Kleine‐Vehn, J. , Ding, Z. , Jones, A.R. , Tasaka, M. , Morita, M.T. and Friml, J. (2010) Gravity‐induced PIN transcytosis for polarization of auxin fluxes in gravity‐sensing root cells. Proc. Natl Acad. Sci. USA 107, 22 344–22 349. 10.1073/pnas.1013145107. 21135243 PMC3009804

[tpj14301-bib-0020] Luschnig, C. , Gaxiola, R.A. , Grisafi, P. and Fink, G.R. (1998) EIR1, a root‐specific protein involved in auxin transport, is required for gravitropism in *Arabidopsis thaliana* . Genes Dev. 12, 2175–2187. 10.1101/gad.12.14.2175. 9679062 PMC317016

[tpj14301-bib-0021] Marhavý, P. , Vanstraelen, M. , De Rybel, B. , Zhaojun, D. , Bennett, M.J. , Beeckman, T. and Benková, E. (2013) Auxin reflux between the endodermis and pericycle promotes lateral root initiation. EMBO J. 32, 149–158. 10.1038/emboj.2012.303. 23178590 PMC3545298

[tpj14301-bib-0022] McDowell, J.M. , An, Y.Q. , Huang, S. , McKinney, E.C. and Meagher, R.B. (1996a) The *Arabidopsis ACT7* actin gene is expressed in rapidly developing tissues and responds to several external stimuli. Plant Physiol. 111, 699–711. 10.1104/pp.111.3.699. 8754679 PMC157885

[tpj14301-bib-0023] McDowell, J.M. , Huang, S. , McKinney, E.C. , An, Y.Q. and Meagher, R.B. (1996b) Structure and evolution of the *actin* gene family in *Arabidopsis thaliana* . Genetics, 142, 587–602. 8852856 10.1093/genetics/142.2.587 PMC1206991

[tpj14301-bib-0024] Michniewicz, M. , Zago, M.K. , Abas, L. ***et al.*** (2007) Antagonistic regulation of PIN phosphorylation by PP2A and PINOID directs auxin flux. Cell, 130, 1044–1056. 10.1016/j.cell.2007.07.033. 17889649

[tpj14301-bib-0025] Morita, M.T. , Saito, C. , Nakano, A. and Tasaka, M. (2007) Endodermal‐amyloplast less 1 is a novel allele of SHORT‐ROOT. Adv. Space Res. 39, 1127–1133. 10.1016/j.asr.2006.12.020.

[tpj14301-bib-0026] Nakamura, M. , Toyota, M. , Tasaka, M. and Morita, M.T. (2011) An *Arabidopsis* E3 ligase, SHOOT GRAVITROPISM9, modulates the interaction between statoliths and F‐actin in gravity sensing. Plant Cell, 23, 1830–1848. 10.1105/tpc.110.079442. 21602290 PMC3123953

[tpj14301-bib-0027] Naramoto, S. , Kleine‐Vehn, J. , Robert, S. ***et al.*** (2010) ADP‐ribosylation factor machinery mediates endocytosis in plant cells. Proc. Natl Acad. Sci. USA 107, 21 890–21 895. 10.1073/pnas.1016260107. PMC3003034 21118984

[tpj14301-bib-0028] Nick, P. , Han, M.J. and An, G. (2009) Auxin stimulates its own transport by shaping actin filaments. Plant Physiol. 151, 155–167. 10.1104/pp.109.140111. 19633235 PMC2736007

[tpj14301-bib-0029] Nishimura, T. , Yokota, E. , Wada, T. , Shimmen, T. and Okada, K. (2003) An *Arabidopsis* ACT2 dominant‐negative mutation, which disturbs F‐actin polymerization, reveals its distinctive function in root development. Plant Cell, Physiol. 44, 1131–1140. 14634149 10.1093/pcp/pcg158

[tpj14301-bib-0030] Okamoto, K. , Ueda, H. , Shimada, T. , Tamura, K. , Kato, T. , Tasaka, M. , Morita, M.T. and Hara‐Nishimura, I. (2015) Regulation of organ straightening and plant posture by an actin–myosin XI cytoskeleton. Nat. Plants, 1, 15 031 10.1038/nplants. 27247032

[tpj14301-bib-0031] Paciorek, T. and Friml, J. (2006) Auxin signaling. J. Cell Sci. 119, 1199–1202. 10.1242/jcs.02910. 16554435

[tpj14301-bib-0032] Rakusová, H. , Gallego‐Bartolome, J. , Vanstraelen, M. , Robert, H.S. , Alabadi, D. , Blázquez, M.A. , Benková, E. and Friml, J. (2011) Polarization of PIN3‐dependent auxin transport for hypocotyl gravitropic response in *Arabidopsis thaliana* . Plant J. 67, 817–826. 10.1111/j.1365-313X.2011.04636.x. 21569134

[tpj14301-bib-0033] Rakusová, H. , Fendrych, M. and Friml, J. (2015) Intracellular trafficking and PIN‐mediated cell polarity during tropic responses in plants. Curr. Opin. Plant Biol. 23, 116–123. 10.1016/j.pbi.2014.12.002. 25553419

[tpj14301-bib-0034] Rakusová, H. , Abbas, M. , Han, H. , Song, S. , Robert, H.S. and Friml, J. (2016) Termination of shoot gravitropic responses by auxin feedback on PIN3 polarity. Curr. Biol. 26, 3026–3032. 10.1016/j.cub.2016.08.067. 27773568

[tpj14301-bib-0035] Su, S.H. , Gibbs, N.M. , Jancewicz, A.L. and Masson, P.H. (2017) Molecular mechanisms of root gravitropism. Curr. Biol. 27, R964–R972. 10.1016/j.cub.2017.07.015. 28898669

[tpj14301-bib-0036] Talts, K. , Ilau, B. , Ojangu, E.L. , Tanner, K. , Peremyslov, V.V. , Dolja, V.V. , Truve, E. and Paves, H. (2016) *Arabidopsis* myosins XI1, XI2, and XIK are crucial for gravity‐induced bending of inflorescence stems. Front. Plant Sci. 7, 1932 10.3389/fpls.2016.01932. 28066484 PMC5174092

[tpj14301-bib-0037] Taniguchi, M. , Furutani, M. , Nishimura, T. ***et al.*** (2017) The *Arabidopsis* LAZY1 family plays a key role in gravity signaling within statocytes and in branch angle control of roots and shoots. Plant Cell, 29, 1984–1999. 10.1105/tpc.16.00575. 28765510 PMC5590491

[tpj14301-bib-0038] Wiśniewska, J. , Xu, J. , Seifertová, D. , Brewer, P.B. , Růžička, K. , Blilou, I. , Rouquié, D. , Benková, E. , Scheres, B. and Friml, J. (2006) Polar PIN localization directs auxin flow in plants. Science, 312, 883 10.1126/science.1121356. 16601151

[tpj14301-bib-0039] de Wit, M. , Galvão, V.C. and Fankhauser, C. (2016) Light‐mediated hormonal regulation of plant growth and development. Annu. Rev. Plant Biol. 67, 513–537. 10.1146/annurev-arplant-043015-112252. 26905653

[tpj14301-bib-0040] Yamamoto, K. and Kiss, J.Z. (2002) Disruption of the actin cytoskeleton results in the promotion of gravitropism in inflorescence stems and hypocotyls of *Arabidopsis* . Plant Physiol. 128, 669–681. 10.1104/pp.010804. 11842170 PMC148928

[tpj14301-bib-0041] Žádníková, P. , Petrášek, J. , Marhavý, P. ***et al.*** (2010) Role of PIN‐mediated auxin efflux in apical hook development of *Arabidopsis thaliana* . Development, 137, 607–617. 10.1242/dev.041277. 20110326

