# Supplementary material for: Genetic screen for factors mediating PIN polarization in gravistimulated Arabidopsis thaliana hypocotyls
Source: Plant J. 2019 Apr 10;98(6):1048–59. doi: 10.1111/tpj.14301 (PMC6618169; doi:10.1111/tpj.14301)
Supplement: Supplementary file 18 — Table S2. List of candidate genes for hrb17 and hhb13 mutants. [file TPJ-98-1048-s018.docx]

**Supplementary Tab 2. Mutated candidate genes of mutant *hrb17* and *hhb13*.**

| **Mutants** | **Chromosome** | **Position** | **Mutation** | **Amino acid change** | **Frequency** | **Gene** | **Description** |
| --- | --- | --- | --- | --- | --- | --- | --- |
| *hrb17* | Chr3 | 17786891 | GAC>AAC | D>N | 0.77 | AT3G48170 | ALDEHYDE DEHYDROGENASE 10A9, [aldehyde dehydrogenase (NAD) activity](https://www.arabidopsis.org/servlets/TairObject?type=keyword&id=1428), [chloroplast](https://www.arabidopsis.org/servlets/TairObject?type=keyword&id=175) |
|  | Chr3 | 20072023 | CAA>TAA | Q>U | 0.85 | AT3G54220 | SCARECROW |
|  | Chr3 | 21823118 | AGT>AAT | S>N | 0.71 | AT3G59040 | Tetratricopeptide repeat (TPR)-like superfamily protein |
|  | Chr3 | 22668387 | TCT>TTT | S>F | 0.80 | AT3G61240 | DEA(D/H)-box RNA helicase family protein |
| *hhb13* | Chr3 | 6476082 | TCT>TTT | S>F | 1.00 | AT3G18780 | ACTIN 2 |
