# Supplementary material for: Genetic screen for factors mediating PIN polarization in gravistimulated Arabidopsis thaliana hypocotyls
Source: Plant J. 2019 Apr 10;98(6):1048–59. doi: 10.1111/tpj.14301 (PMC6618169; doi:10.1111/tpj.14301)
Supplement: Supplementary file 19 [file TPJ-98-1048-s019.docx]

**Supporting Information**

**Figure S1. Schemes representing cellular membranes used for PIN3-GFP/YFP quantification.**

(a) Regions of interest for quantification of auxin-induced PIN3-GFP/YFP repolarization. Red line: cortex cell; blue line: outer side of endodermal cells; pink line: inner side of endodermal cells; white square area: vasculature. Scale bar = 20 µm. Arrow indicates gravity direction.

(b) Regions of interest for quantification of gravity-induced PIN3-GFP repolarization. Blue line: outer side of endodermal cells at lower and upper side of hypocotyl. Scale bar = 20 µm. Arrow indicates gravity direction.

**Figure S2. Gravity-induced PIN3-GFP relocation in *hrb* and *hhb* mutants.**

(a) PIN3-GFP in *hrb1* and *hrb2* mutants after 6 hours gravity stimulation. Scale bars = 20 µm. Arrowheads depict the outer sides of endodermal cells. Arrow indicates gravity direction.

(b) Quantification of PIN3-GFP signal in *hrb1* and *hrb2* mutants after 6 hours gravity stimulation. PIN3-GFP fluorescence was compared between outer side of endodermal cells at lower and upper side of hypocotyl. Error bars are SE. (N > 15 seedlings for each replicate, Student’s t-test, ** *P* < 0.05).

(c) PIN3-GFP in *hhb4* and *hhb12* mutants after 24 hours gravity stimulation. Scale bars = 20 µm. Arrowheads depict the outer sides of endodermal cells. Arrow indicates gravity direction.

(d) Quantification of PIN3-GFP signal in *hhb4* and *hhb12* mutants after 24 hours gravity stimulation. PIN3-GFP fluorescence was compared between outer side of endodermal cells at lower and upper side of hypocotyl. Error bars are SE. (N > 15 seedlings for each replicate, Student’s t-test, ** *P* < 0.05).

**Figure S3. Auxin-induced PIN3-GFP inner-lateralization in *hrb* and *hhb* mutants.**

(a) 4 hours DMSO or NAA treated *SCR::PIN3-YFP* seedlings . Scale bars = 20 µm. Arrowheads depict the outer sides of endodermal cells.

(b) Quantification of PIN3-YFP signal after 4 hours NAA treatment. PIN3-YFP fluorescence was compared between inner and outer side of endodermal cells. Error bars are SE. (N > 15 seedlings for each replicate, Student’s t-test, ** *P* < 0.05).

(c) Quantification of PIN3-GFP signal in vasculature after 4 hours DMSO or NAA treatment. (N = 25)

(d) PIN3-GFP in *hrb1* and *hrb2* mutants after 4 hours DMSO or NAA treatment. Scale bars = 20 µm. Arrowheads depict the outer sides of endodermal cells.

(e) Quantification of PIN3-GFP signal in *hrb1* and *hrb2* mutants. PIN3-GFP fluorescence was compared between inner and outer side of endodermal cells. Error bars are SE. (N > 15 seedlings for each replicate, Student’s t-test, ** *P* < 0.05)

(f) PIN3-GFP in *hhb2* and *hhb3* mutants after 4 hours of DMSO or NAA treatment. Scale bars = 20 µm. Arrowheads depict the outer sides of endodermal cells.

(g) Quantification of PIN3-GFP signal in *hhb2* and *hhb3* mutants. PIN3-GFP fluorescence was compared between inner and outer side of endodermal cells. Error bars are SE. (N > 15 seedlings for each replicate, Student’s t-test, ** *P* < 0.05).

**Figure S4. Multiple PIN-related phenotype in the *hrb* and *hhb* mutants.**

(a) Image of 24 hours gravity stimulated wild type, *hrb2* and *hhb9* mutants. Arrow indicates gravity direction. Scale bar = 2 cm.

(b) Image of phototropic response of wild type, *hrb2* and *hhb9* mutants after 24 hours illumination by unilateral light. Arrow indicates light source direction. Scale bar = 2 cm.

(c) Lateral root development in 7 days old wild type and *hrb2* mutant. Scale bar = 2 cm.

(d) Defective apical hook maintenance in *hrb2* mutant. Wild type and *hrb2* seeds were grown under light for 16 hours, then covered for another 48 hours. Scale bar = 20 µm.

(e) Quantification of gravitropic curvature of wild type, *hhb9* and *hrb2* mutants after 24 hours gravistimulation. Error bars are SE. (N > 25 seedlings for each replicate, Student’s t test, ** *P* < 0.05).

(f) Quantification of phototropic curvature of wild type, *hhb9* and *hrb2* mutants after 24 hours illumination by unilateral light. Error bars are SE. (N > 20 seedlings for each replicate, Student’s test t-test, ** *P* < 0.05).

(g) Images of apical hook of wild type and *hrb2* mutant at 32 hours and 48 hours. Scale bar = 1 cm.

(h) Quantification of apical hook angle at different time points of wild type and *hrb2* mutant. Error bars are SE. (N = 15, Student’s t-test between wild type and *hrb2* mutant, ** *P* < 0.05).

**Figure S5. Plots of mutation frequency in the genome of *hrb17* and *hhb13* mutants.**

Each plot of mutation’s frequencies represents one chromosome of mutant *hrb17* (a) and *hhb13* (b). Green squares mark possible intervals with increased mutation frequency on the chromosome. Mutations common with the background line *PIN3::PIN3-GFP* were subtracted.

**Figure S6. Allelic test of *hrb17* mutant.**

(a) Propidium iodide staining of 3 days etiolated hypocotyls of wild type, *scr-3* and *hrb17* mutants. Arrowheads depict the endodermal cells. Scale bar = 20 µm. (N = 10).

(b) Representative image of wild type and F1 seedlings of *hrb17* × *scr-3* after 24 hours after gravity stimulation. Scale bar = 1 cm. Arrowheads indicate gravity direction.

(c) Quantification of hypocotyl bending angle of wild type, and F1 seedlings of *hrb17* × *scr-3* after 24 hours after gravity. Error bars are SE. (N = 20, Student’s t-test, ** *P* < 0.05).

(d) Quantification of root bending angle of wild type, *hrb17*, *scr-3* and F1 seedlings of *hrb17* × *scr-3* after 24 hours after gravity. Error bars are SE. (N = 20, Student’s t-test, ** *P* < 0.05).

**Figure S7. Starch staining in wild type and *hrb17* mutant.**

3 days old etiolated seedlings grown vertically, seedlings were stained with Lugol solution for 3 minutes. The *hrb17* mutant (b) shows less starch as compared to wild type (a). N = 15 seedlings for each replicate. Scale bar = 1 cm. Arrowheads indicate starch. Arrow indicates gravity direction.

**Figure S8. Auxin-induced PIN3-GFP relocation in cortical cell.**

**(**a) Cortex cells after 4 hours DMSO or NAA treatment. No visible auxin-induced PIN3-GFP relocation in the cortex. Arrowheads depict PIN3 at outer cell layer of cortex cell. Scale bar = 20 µm.

(b) Quantification of PIN3-GFP signal at outer layer of cortex cells. Error bars are SE. (N > 15 seedlings for each replicate).

**Figure S9. Gravity-induced PIN3 polarization in *eal1*/*shr* mutant.**

(a) PIN3-GFP in *eal1/shr* mutant without gravity stimulation. Scale bar = 20 µm.

(b) PIN3-GFP in *eal1/shr* mutant after 6 hours gravity stimulation. Scale bar = 20 µm.

(c) PIN3-GFP in *eal1/shr* mutant after 24 hours gravity stimulation. Scale bar = 20 µm.

Arrowheads depict the outer sides of endodermal cells. Arrow indicates gravity direction.

(d - e) Quantification of PIN3-GFP signal in *eal1*/*shr* mutant after 6 hours (d) and 24 hours (e) gravity stimulation. PIN3-GFP fluorescence was compared between outer side of endodermal cells at lower and upper side of hypocotyl. Error bars are SE. (N > 15 seedlings for each replicate, Student’s t-test, ** *P* < 0.05).

**Figure S10. Auxin-induced PIN3 polarization in *eal1*/*shr* mutant.**

(a) PIN3-GFP in *eal1/shr* mutant after 4 hours DMSO or NAA treatment. Scale bar = 20 µm. Arrowheads depict the outer sides of endodermal cells.

(b) Quantification of PIN3-GFP signal in *eal1*/*shr* mutant. PIN3-GFP fluorescence was compared between inner and outer side of endodermal cells. Error bars are SE. (N > 15 seedlings for each replicate, Student’s t-test, ** *P* < 0.05).

**Figure S11. Bending angle of Col, *actin2*, *actin7* and *actin8* and higher-order *actin* mutants.**

3 day etiolated seedlings were gravistimulated for 24 hours in single *actin2, actin7* and *actin8* mutant and higher-order mutants. Error bars are SE. (N = 25 - 40 seedlings for each replicate, Student’s t-test, ** *P* < 0.05).

**Figure S12. Gravity-induced PIN3 polarization in *actin2* mutant.**

(a) PIN3-GFP in *actin2* mutant without gravity stimulation. Scale bar = 20 µm.

(b) PIN3-GFP in wild type after 6 hours gravity stimulation. Scale bar = 20 µm.

(c) PIN3-GFP in *actin2* mutant after 6 hours gravity stimulation. Scale bar = 20 µm.

(d) PIN3-GFP in wild type after 24 hours gravity stimulation. Scale bar = 20 µm.

(e) PIN3-GFP in *actin2* mutant after 24 hours gravity stimulation. Scale bar = 20 µm.

Arrowheads depict the outer sides of endodermal cells. Arrow indicates gravity direction.

(f - g) Quantification of PIN3-GFP signal after 6 hours (f) and 24 hours (g) gravity stimulation. PIN3-GFP fluorescence was compared between outer side of endodermal cells at lower and upper side of hypocotyl. Error bars are SE. (N > 15 seedlings for each replicate, Student’s t-test between, ** *P* < 0.05).

**Figure S13. Auxin-induced PIN3 polarization in *actin2* mutant.**

(a) PIN3-GFP in *actin2* mutant after 4 hours DMSO or NAA treatment. Scale bar = 20 µm. Arrowheads depict the outer sides of endodermal cells.

(b) Quantification of PIN3-GFP signal after 4 hours NAA treatment. PIN3-GFP fluorescence was compared between inner and outer side of endodermal cells. Error bars are SE. (N > 15 seedlings for each replicate, Student’s t-test, ** *P* < 0.05).

**Figure S14. LatB and Oryzalin inhibits hypocotyl growth.**

3 day etiolated seedlings were transferred onto new plates with DMSO, 2 µM, 5 µM, 10 µM and 30 µM of LatB and Oryzalin, respectively. Seedlings were kept growing in the dark for 24 hours and hypocotyl length was measured. Error bars are SE. **(**N = 25 - 40 seedlings for each replicate**,** Student’s t-test, ** *P* < 0.05).

**Figure S15. Oryzalin inhibits gravity-induced PIN3 relocation.**

(a) DMSO-treated or Oryzalin-treated PIN3-GFP seedlings after 6 hours gravistimulation. Scale bar = 20 µm. Arrowheads depict the outer sides of endodermal cells. Arrow indicates gravity direction.

(b) Quantification of PIN3-GFP signal upon Oryzalin treatment after 6 hours gravity stimulation. PIN3-GFP fluorescence was compared between outer side of endodermal cells at lower and upper side of hypocotyl. Error bars are SE. (N > 15 seedlings for each replicate, Student’s t-test, ** *P* < 0.05).

**Figure S16. LatB does not affect gravity-induced PIN3 relocation.**

(a) DMSO treated PIN3-GFP seedlings after 6 hours gravistimulation. Scale bar = 20 µm.

(b) LatB treated PIN3-GFP seedlings after 6 hours gravistimulation. Scale bar = 20 µm.

(c) DMSO treated PIN3-GFP seedlings after 24 hours gravistimulation. Scale bar = 20 µm.

(d) LatB treated PIN3-GFP seedlings after 24 hours gravistimulation. Scale bar = 20 µm. Arrowheads depict the outer sides of endodermal cells. Arrow indicates gravity direction.

(e - f) Quantification of PIN3-GFP signal after 6 hours (e) and 24 hours (f) gravistimulation. PIN3-GFP fluorescence was compared between outer side of endodermal cells at lower and upper side of hypocotyl. Error bars are SE. (N > 15 seedlings for each replicate, Student’s t-test, ** *P* < 0.05).

**Table S1. Phenotype analysis of 37 candidate mutants from the forward genetic screen.**

**Table S2. List of candidate genes for *hrb17* and *hhb13* mutants.**
